# Genes Involved in the Metabolism of Poly-Unsaturated Fatty-Acids (PUFA) and Risk for Crohn's Disease in Children & Young Adults

**DOI:** 10.1371/journal.pone.0015672

**Published:** 2010-12-20

**Authors:** Irina Costea, David R. Mack, David Israel, Kenneth Morgan, Alfreda Krupoves, Ernest Seidman, Colette Deslandres, Philippe Lambrette, Guy Grimard, Emile Levy, Devendra K. Amre

**Affiliations:** 1 Public Health Agency of Canada, Montreal, Canada; 2 Research Centre, Sainte-Justine Hospital, Montreal, Canada; 3 Division of Gastroenterology, Hepatology and Nutrition, Children's Hospital of Eastern Ontario, Ottawa, Canada; 4 Department of Gastroenterology, Hepatology and Nutrition, British Columbia's Children's Hospital, Vancouver, Canada; 5 Department of Human Genetics, McGill University and the Research Institute of the McGill University Health Center, Montreal, Canada; 6 Department of Preventive and Social Medicine, University of Montreal, Montreal, Canada; 7 Department of Medicine, McGill University and the Research Institute of the McGill University Health Center, Montreal, Canada; 8 Department of Pediatrics, University of Montreal, Montreal, Canada; 9 Division of Orthopedics, Department of Pediatrics, University of Montreal, Montreal, Canada; 10 Department of Nutrition, University of Montreal, Montreal, Canada; Ludwig Maximilian University of Munich, Germany

## Abstract

**Background and Objectives:**

Epidemiological evidence for the role of polyunsaturated fatty-acids (PUFA) in Crohn's disease (CD) is unclear, although the key metabolite leucotriene B4 (LTB_4_) is closely linked to the inflammatory process. We hypothesized that inherited variation in key PUFA metabolic enzymes may modify susceptibility for CD.

**Methods and Principal Results:**

A case-control design was implemented at three pediatric gastroenterology clinics in Canada. Children ≤20 yrs diagnosed with CD and controls were recruited. 19 single nucleotide polymorphisms (SNPs) across the *ALOX5* (4) *CYP4F3* (5) and *CYP4F2* (10) genes, were genotyped. Associations between SNPs/haplotypes and CD were examined. A total of 431 cases and 507 controls were studied. The mean (±SD) age of the cases was 12.4 (±3.3) years. Most cases were male (56.4%), had ileo-colonic disease (L3±L4, 52.7%) and inflammatory behavior (B1±p, 87%) at diagnosis. One genotyped *CYP4F3* SNP (rs2683037) not in Hardy-Weinberg Equilibrium was excluded. No associations with the remaining 4 *CYP4F3* SNPs with CD were evident. However haplotype analysis revealed associations with a two-marker haplotype (TG) (rs3794987 & rs1290617) (p = 0.02; permuted p = 0.08). *CYP4F2* SNPs, rs3093158 (OR (recessive) = 0.56, 95% CI = 0.35–0.89; p = 0.01), rs2074902 (OR (trend) = 1.26, 95% CI = 1.00–1.60; p = 0.05), and rs2108622 (OR (recessive) = 1.6, 95% CI = 1.00–2.57; p = 0.05) were significantly associated whereas rs1272 (OR (recessive) = 0.58, 95% CI = 0.30–1.13; p = 0.10) showed suggestions for associations with CD. A haplotype comprising these 4 SNPs was significantly associated (p = 0.007, permuted p = 0.02) with CD. Associations with SNP rs3780901 in the *ALOX5* gene were borderline non-significant (OR (dominant) = 1.29, 95% CI = 0.99–1.67; p = 0.056). A haplotype comprising the 4 *ALOX5* SNPs (TCAA, p = 0.036) was associated with CD, but did not withstand corrections for multiple comparisons (permuted p = 0.14).

**Conclusions:**

Inherited variation in enzymes involved in the synthesis/metabolism of LTB_4_ may be associated with CD. These findings implicate PUFA metabolism as a important pathway in the CD pathogenesis.

## Introduction

Crohn's disease (CD) a chronic inflammatory bowel disease (IBD) is common in children and appears to be on the rise in most developing countries including Canada [1], [2]. Children with CD phenotypically differ from adults with CD and present unique clinical challenges relative to their more aggressive disease [3]–[5].

Recent genome-wide association (GWA) studies both in adults and children [6]–[9] have provided valuable insights on the potential mechanisms that underlie the chronic inflammation that is characteristic of CD. Much however remains to be known as GWA studies have accounted for <20% of the inherited variation in CD [7]. CD may represent a group of heterogeneous diseases with unique and overlapping pathophysiologies and in this context, it remains to be explored how environmental factors modify the expression of CD among genetically susceptible individuals.

We have recently shown that in Canadian children an imbalance in consumption of dietary polyunsaturated fatty acids (PUFA) may be associated with risk for CD [10]. In particular the consumption ratio of ω6/ω3 PUFA was observed to be of relevance, as has been proposed by others [11], [12]. However, many epidemiological studies [13] and clinical trials [14] using sources of ω fatty acids have not provided consistent results. We hypothesized that inherited variation in the ability to metabolize dietary PUFA may mediate development of CD and may have contributed to the previously observed inconsistent results.

The PUFA metabolic pathway is a complex pathway involving interplay of various enzymes [15]. Key steps relate to the release of arachidonic acid, a ω-6 fatty acid from the cell walls and synthesis of inflammatory mediators known as eicosanoids. A key eicosanoid is leucotriene-B4 (LTB_4_), a well recognized mediator of inflammation. Indeed, various studies have shown that LTB_4_ levels are associated with CD inflammation [16], [17]. Interestingly, a recent study has shown that the levels and activities of various enzymes involved in the PUFA metabolic pathway that leads to production of LTB_4_ were related to inflammation in IBD [18]. Three key enzymes are the 5-LO (5-lipoxygenase) that metabolizes arachidonic acid and initiates the pathway and the cytochrome P450 enzymes, CYP4F3 and CYP4F2 that are known de-activators of LTB_4_ [19]. In this study we investigated whether DNA variations in these key genes were associated with CD in children.

## Methods

### Ethics statement

Ethical approval was acquired from the Ethics Review Board of the Ste-Justine Hospital Foundation (HSJ), Montreal; the Children's Hospital of Eastern Ontario (CHEO), Ottawa; and the British Columbia's Children's Hospital, Vancouver. Informed written consent was obtained from all participants (directly from the subject if he/she was an adult or from the parent/guardian if otherwise).

A case-control study was carried out. Cases were children (≤20 yrs of age) diagnosed with CD and recruited from 3 pediatric gastroenterology clinics across Canada (Montreal, Ottawa, Vancouver). Diagnosis of CD was based on established criteria that included clinical, radiological, endoscopic and histological confirmation [20], [21]. Disease location and behavior were classified according to the Montreal Classification [22]. Controls were recruited from various sources to parallel population representativeness. These included children visiting the orthopedic clinics for minor trauma (fractures mostly), population-based controls (children) identified using random digit dialing, a birth cohort and a cohort of healthy adults recruited for ongoing genetic epidemiology studies at the Montreal study center. Cases and controls were restricted to those with self-reported European ancestry. Most of these controls have been previously utilized to replicate/validate recent associations reported either in candidate gene or GWA studies [23]–[28]. Blood and/or saliva were collected as a source for DNA.

### Selection of markers, genotyping & statistical analysis

Three genes, *ALOX5*, *CYP4F3* and *CYP4F2* were selected for study as they regulate critical *upstream* and *downstream* events that lead to production/metabolism of LTB_4_. Relevant markers to genotype were identified using the tag-SNP approach [29]. The following parameters were employed: linkage disequilibrium (LD)>0.80, minor allele frequencies >10%. Genotyping data for populations of European origin housed at the Seattle SNPs data resources (http://gvs.gs.washington.edu/GVS) was utilized to select the tag-SNPs. SNPs were genotyped using the Sequenom platform at the McGill University & Genome Quebec Innovation Center in Montreal. Primers utilized for genotyping the SNP are listed in table S1. Prior to analysis, Hardy-Weinberg equilibrium (HWE) was examined in the controls. Allelic, genotype and haplotype analysis was carried out using PLINK http://pngu.mgh.harvard.edu/~purcell/plink) and HAPLOVIEW (http://broad.mit.edu/mpg/HAPLOVIEW). Various models of inheritance (additive, dominant, and recessive) were investigated. Odds ratios (OR) and corresponding 95% confidence intervals (95% CI) were estimated. P-values for the haplotype analysis were corrected using permutation (n = 10000).

## Results

A total of 431 cases and 507 controls were investigated (table 1). There were more males and the most common location of disease was the ileo-colonic site (L3±L4). Most children had inflammatory behavior at diagnosis (B1±p).

**Table 1:** Clinical and demographic characteristics of the CD patients.

Characteristic		Cases (N = 431)
Age at diagnosis (Mean (±SD))		12.4 (±3.3)
Gender (%)	Females	188 (43.6)
	Males	243 (56.4)
Disease location (%)†	L1±L4	77 (17.9)
	L3±L4	227 (52.7)
	L2±L4	123 (28.5)
	Only L4	4 (0.93)
Disease behaviour (%)†	B1±p	375 (87.0)
	B2±p	28 (6.5)
	B3±p	28 (6.5)

† Disease location (L1 = isolated ileal; L2 = isolated colonic; L3 = ileo-colonic; L4 = upper tract) and behaviour (B1 = inflammatory; B2 = stricturing; B3 = penetrating; p = perianal disease) was classified at diagnosis, according to WGO's Montreal classification.

Four SNPs in the *ALOX5* gene, 5 in the *CYP4F3* gene and 10 in the *CYP4F2* gene were examined (table S1). SNP rs2683037 in the *CYP4F3* gene was not in HWE in the controls and was excludedfrom further analysis. The average genotype rate was >95%. Single SNP analysis did not reveal associations with the *CYP4F3* gene (table 2). An haplotype analysis however revealed that the 4 markers were distributed in two blocks (table 3) of LD. A two-marker haplotype (block 1) comprising SNPs rs394987 & rs1290617 was significantly associated with CD (p = 0.02). This association however was borderline non-significant on permutation testing (permuted p = 0.08).

**Table 2:** Associations between the *CYP4F3* gene and CD in Canadian children.

SNP	Model	Cases	Controls	P-value
rs3794987 (C/T)	TREND	431/453	456/514	0.46
	DOM	322/120	347/138	0.66
	REC	109/333	109/376	0.43
rs1290617 (G/A)	TREND	314/560	373/593	0.23
	DOM	255/182	301/182	0.22
	REC	59/378	72/411	0.54
rs2283612 (T/G)	TREND	393/487	404/562	0.22
	DOM	305/135	320/163	0.32
	REC	88/352	84/399	0.31
rs4646904 (C/T)	TREND	160/726	173/801	0.86
	DOM	145/298	162/325	0.86
	REC	15/428	11/476	0.29

DOM: dominant, REC: recessive, TREND: Cochran-Armitage trend test. For this test, the numbers in the table represent the number of chromosomes that had the minor or the major allele.

**Table 3:** Association between *CYP4F3* haplotypes and CD.

Blocks	Haplotype	Case	Control	P-value
Block 1	CG	0.51	0.52	0.55
	TA	0.36	0.37	0.44
	TG	0.13	0.10	0.02* †
Block 2	TC	0.55	0.58	0.24
	GC	0.27	0.24	0.23
	GT	0.18	0.17	0.82

Block 1 (rs3794987, rs1290617); Block 2 (rs2283612, rs4646904);

*p-value<0.05;

† permuted p-value = 0.08.

For the *ALOX5* gene, there were suggestions for associations with one SNP rs3780901 (OR = 1.29, 95% CI = 0.99–1.67; p = 0.056) under a dominant model (table 4). Haplotype analysis (table 5) comprising the 4 SNPs revealed 5 high frequency (>5%) haplotypes of which haplotype TCAA was significantly associated with CD (p = 0.036). The latter associations however did not withstand corrections for multiple comparisons (permuted p = 0.14).

**Table 4:** Associations between the *ALOX5* gene and CD in Canadian children.

SNP	Model	Cases	Controls	P-value
rs2115819 (C/T)	TREND	394/444	437/535	0.39
	DOM	296/123	334/152	0.53
	REC	98/321	103/383	0.43
rs3780901 (C/T)	TREND	256/592	324/652	0.17
	DOM	213/211	276/212	0.056
	REC	43/381	48/440	0.88
rs2291427 (A/G)	TREND	249/599	314/662	0.20
	DOM	209/215	264/224	0.15
	REC	40/384	50/438	0.68
rs10751383 (A/C)	TREND	349/495	435/541	0.17
	DOM	273/149	337/151	0.16
	REC	76/346	98/390	0.43

DOM: dominant, REC: recessive, TREND: Cochran-Armitage trend test. For this test, the numbers in the table represent the number of chromosomes that had the minor or the major allele.

**Table 5:** Associations between *ALOX5* haplotypes and CD.

Haplotype	Cases (%)	Controls (%)	P-value
CTGC	0.30	0.29	0.64
TCAA	0.16	0.20	0.036* †
TTGA	0.15	0.15	0.75
TTGC	0.14	0.13	0.52
CTAC	0.06	0.05	0.39

Haplotypes comprising SNPs: rs2115819, rs3780901, rs2291427, and rs10751383;

*p-value<0.05;

† permuted p-value = 0.14.

Of the ten SNPs in the *CYP4F2* gene investigated, three were significantly associated with CD (table S2) under various models of inheritance. SNP rs3093158 showed protective associations with CD under the recessive model (OR = 0.56, 95% CI = 0.35–0.89; p = 0.01), SNP rs2074902 was risk conferring under the additive model (OR = 1.26, 95% CI = 1.00–1.60; p = 0.05), and SNP rs2108622 was risk conferring under the recessive model (OR = 1.6, 95% CI = 1.00–2.57; p = 0.05). There were suggestions for associations with a fourth SNP rs1272 under the recessive models (p = 0.10) but these did not achieve nominal statistical significance. Haplotype associations (table 6) using the 4 *CYP4F2* SNPs (rs1272, rs3093158, rs2072902, and rs2108622) indicated significant associations with haplotype GGTC (p = 0.007) and marginally non-significant associations with haplotype GACT (p = 0.06). Associations with haplotype GGTC remained significant (permuted p = 0.02) after corrections for multiple comparisons.

**Table 6:** Associations between *CYP4F2* haplotypes and CD.

Haplotype	Cases (%)	Controls (%)	P-value
GATC	0.36	0.36	0.93
CGTC	0.21	0.21	0.84
GACT	0.21	0.18	0.06
GATT	0.12	0.13	0.81
GGTC	0.09	0.13	0.007* †

Haplotypes comprising SNPs rs1272, rs3093158, rs2074902, and rs2108622;

*P-value<0.05,

† permuted p-value = 0.02.

## Discussion

In this study we examined whether key enzymes within the fatty-acid metabolic pathway were associated with risk for CD. Although there were suggestions for associations with the *ALOX5* gene at the single marker and haplotype level they did not withstand corrections for multiple comparisons. Similarly for the *CYP4F3* gene associations were evident at the haplotype level that were borderline non-significant on correction for multiple comparisons. Associations were evident, both at the single marker and haplotype level, with the *CYP4F2* gene, suggesting that variation in this gene may influence risk for CD in children.

PUFA have long been implicated in the pathogenesis of CD. Various epidemiological investigations have been undertaken to examine whether dietary consumption of PUFA was associated with CD [13]. While some studies suggest that ω-3 PUFA may be beneficial and that a higher ratio of ω-3/ω-6 may be associated with lower risks, evidence across studies has been inconsistent. Similarly, potential benefits from dietary supplementation of PUFA, have been equivocal. As individuals will differ in their capacities to metabolize dietary PUFA, we hypothesized that DNA variation in key genes that metabolize PUFA may be important and may modify any associations between dietary PUFA and CD. An important metabolite of PUFA metabolism is the generation of LTB_4_ a potent mediator of inflammation. It is generated via the metabolism of arachidonic acid, an ω-6 PUFA which is the first metabolite released from the cell membranes. It is a substrate of the 5-LO enzyme coded by the *ALOX5* gene. LTB_4_ derived from the 5-LO metabolic pathway is deactivated mainly by the CYP4F3 (expressed mainly in neutrophils) and CYP4F2 (expressed largely in the liver and kidney) enzymes. We thus selected these three genes for study given their key roles in the metabolic pathway.

The *CYP4F2* & *CYP4F3* genes are located in a cluster on chromosome region 19p13.2. Three genome scans have reported significant linkages to this region in different populations. Rioux et al (2000) [30] using affected sib-pairs reported peak LOD scores of 4.6 for IBD and 3.0 for CD in a Canadian population. In their recent meta-analyses of 10 IBD genome scans among affected relatives, van Heel et al (2004) [31] reported significant linkage of CD to chromosome 19. Low et al (2004) [32] carried out a linkage scan among UK Caucasians (affected sib-pairs) and confirmed linkage of CD to the 19p13.2 region (peak multi-point linkage score of 1.59). The location of the *CYP4F2* & *CYP4F3* genes in the region of significant linkage adds further support to our observations that they may be important candidate genes for CD.

A number of previous studies have implicated LTB_4_ in the pathogenesis of IBD. The greatly enhanced mucosal synthesis of LTB_4_ in IBD [16], [17] or in rectal diasylate [33], [34] of IBD patients has been related, in part, to the increased infiltration of neutrophils into the intestinal tissues [35]. Of relevance to our findings are observations that the metabolism of LTB_4_ by omega-hydroxylase is altered in the colonic mucosa of IBD patients [36], [37]. In particular, the decreased activity of the omega-hydroxylases suggested that the increased, persistent and recurrent inflammation that is characteristic of IBD may be the consequence of an inherent defect in the metabolism of LTB_4_, leading to its enhanced accumulation and activity. It is to be noted however that the CYP4F3 enzyme is the major LTB_4_ omega-hydroxylase [19], [38] with higher detoxification potential as compared to CYP4F2. It would thus have been anticipated that variations in the *CYP4F3* gene were more likely to influence CD susceptibility. Our findings however indicate that the *CYP4F2* gene was more strongly associated with CD than *CYP4F3*. As the tag-SNPs we selected were of high frequency (>10%) it is possible that less frequent SNPs in the *CYP4F3* may be implicated in CD. For example SNP rs28371536 is a non-synonymous *CYP4F3* SNP of low frequency (2%) not investigated in our study. Certainly larger studies will be required to capture less frequent variation in the *CYP4F3* gene and to assess its influence in CD. On the other hand however, it can be speculated that the stronger associations with the *CYP4F2* gene may be related to observations that the enzyme is not only the major hepatic deactivator of LTB_4_ but is also expressed in the small intestine [39] (although at lower levels compared to the liver) and hence would be expected to contribute to LTB_4_ metabolism at the site of inflammation as well. This dual capacity may have contributed to the observed stronger associations. Certainly further studies are required to clarify the roles of both *CYP4F2* and *CYP4F3* genes in CD pathogenesis.

Based on reported observations of elevated levels of LTB_4_ in IBD and CD patients, some studies have examined the expression of the enzymes/proteins involved in LTB_4_ synthesis in IBD. In one study, Hendel et al (2002) [40] reported elevated trends in the levels of 5-LO mRNA when studying 21 CD patients and 12 healthy controls. More recently Jupp et al (2007) [18], in a comprehensive examination of LTB_4_ synthesis pathway enzymes reported a 3-fold higher number of cells staining for 5-LO, a 7-fold higher number of cells staining for FLAP and a 4-fold higher number of cells staining for LTA_4_H in colonic biopsies of patients with active IBD as compared to healthy controls. Although not withstanding corrections for multiple comparisons, nominal associations between the *ALOX5* gene (that codes for the 5-LO enzyme) were evident in our study. These observations highlight that an abnormal prevalence of enzymes that co-ordinate to synthesize LTB_4_ may be intimately linked to tissue injury and inflammation in CD and that this pathway needs to be further investigated.

To our knowledge no previous candidate-gene study has examined associations between the *CYP4F2* gene and CD. Recently, Tello-Ruiz et al (2006) [41] screened variation across the 19p IBD6 locus for associations with IBD. The SNP panel however did not comprise variants in the *CYP4F2* gene. In the same study the authors examined 5 SNPs in the *CYP4F3* gene (including SNP rs1290617 that was part of our panel) but did not find associations with them. Support for a potential role for the *CYP4F2* and *CYP4F3* genes in IBD pathogenesis comes from observations by Curley et al (2006) [42] who found associations between the genes and celiac disease, a disease that bears an inflammatory phenotype similar to CD. On the other hand associations with CD for the three genes examined in this study were not noted in earlier GWA studies, at the genome-wide significance level implemented. However, in the recent GWA study on ulcerative colitis (UC) [43], strong associations between SNPs within the *PLA2G2E* (phospholipase A2, group 2E) locus were noted in a North American Caucasian cohort. The *PLA2G2E* gene codes for a secretory phospholipase A2 that releases arachidonic acid from the cell membrane. The enzyme has been shown to be involved in the synthesis of leucotrienes and participate in the inflammatory process [44]. Considering that UC and CD share pathogenetic features, the gene would be a prime candidate gene for study in CD susceptibility and is currently being investigated in our cohort.

It is interesting to note that of the *CYP4F2* associated SNPs, SNP rs2108622 is a non-synonymous coding SNP in exon 11 (M433V). eQTL analysis [45] indicated that the coding variation leads to significant alteration in mRNA expression (LOD score = 3.15, p-value = 0.00014). Similarly, SNP rs2074902 an intronic SNP is in perfect LD with SNP rs3093105, a non-synonymous coding SNP (W12G). Although eQTL data for rs3093105 was not available, using rs2074902 as a proxy indicated that the variation could alter mRNA expression significantly (LOD score = 2.905, p-value = 0.00025). Taken together these findings suggest that altered expression of the *CYP4F2* gene may be related to de-regulation of LTB_4_ metabolism that in turn can modify the inflammatory responses.

In conclusion, our findings suggest that DNA variation in the metabolism of LTB_4_ is associated with risk for CD in children. Larger studies to replicate findings in independent cohorts and functional studies to determine biological mechanisms are required. Furthermore studies to investigate other potential candidate genes in the pathway (viz. *FLAP*, *PLA2G2E*, *LTA4H*, *LTB4R1* etc.) need to be carried out. In addition, investigation of interactions between dietary consumption of fatty acids and PUFA pathway metabolic genes vis-à-vis risk for CD need to be pursued.

## Supporting Information

Table S1 Primers used for genotyping the *ALOX5*, *CYP4F3* and *CYP4F2* SNPs.
(DOC)

Table S2 Associations between the *CYP4F2* gene and risk for CD in Canadian children.
(DOC)
