# Supplementary material for: Genes Involved in the Metabolism of Poly-Unsaturated Fatty-Acids (PUFA) and Risk for Crohn's Disease in Children & Young Adults
Source: PLoS One. 2010 Dec 20;5(12):e15672. doi: 10.1371/journal.pone.0015672 (PMC3004960; doi:10.1371/journal.pone.0015672)
Supplement: Table S1 — Primers used for genotyping the ALOX5, CYP4F3 and CYP4F2 SNPs. (DOC) [file pone.0015672.s001.doc]

| **Gene** | **SNP** | **Strand** | **Primers** |
| --- | --- | --- | --- |
| *ALOX5* | rs2115819 | F | ACGTTGGATGTGTTGCTTTTGCCACAGGAG |
|  |  | R | ACGTTGGATGTTTGTGTAACACTGGGATGG |
|  | rs3780901 | F | ACGTTGGATGAGAGGCCTTTGTGAGTACTG |
|  |  | R | ACGTTGGATGGGCAACTTCCAGCAGTACAC |
|  | rs2291427 | F | ACGTTGGATGGCTCCTAGCTGTTTTCTTCC |
|  |  | R | ACGTTGGATGGATCACTGACATCCCACAGG |
|  | rs10751383 | F | ACGTTGGATGTGAGCTTGTGAGTGTGTCTG |
|  |  | R | ACGTTGGATGTGTTGTGTGCTCAGGTGAGG |
| *CYP4F3* | rs3794987 | F | ACGTTGGATGCTGACTTTGGTCAGTACTGC |
|  |  | R | ACGTTGGATGTCTTAGGAGCCCTCATTGTC |
|  | rs1290617 | F | ACGTTGGATGATTCTCCATTGCTGCCCAAG |
|  |  | R | ACGTTGGATGCAGCCTGAATCATTAGGACG |
|  | rs2283612 | F | ACGTTGGATGAATGAGTCCCTGGAAACCTC |
|  |  | R | ACGTTGGATGACTGAGCAGGGAAATCAGAC |
|  | rs4646904 | F | ACGTTGGATGTGTACCACCTTGCAAAGCAC |
|  |  | R | ACGTTGGATGCCTGCACTCACCATTCAATC |
|  | rs2683037 | F | ACGTTGGATGAGAAAGAGAGAGGGCACCAG |
|  |  | R | ACGTTGGATGTCTCCCTGGAGACATTTGAG |
| *CYP4F2* | rs1272 | F | ACGTTGGATGCACATACCACGAAATTCACC |
|  |  | R | ACGTTGGATGGGGATGGTGAAAATGTTCCG |
|  | rs2074900 | F | ACGTTGGATGATCTCTTTAGGCTCACGGTC |
|  |  | R | ACGTTGGATGAGTGGTCTCTCCTGGGTCCT |
|  | rs3093158 | F | ACGTTGGATGTCCCTTCCTCAATCACCTTC |
|  |  | R | ACGTTGGATGGTAGAAGGGAGCTTCATGTG |
|  | rs2074902 | F | ACGTTGGATGTACGAGGCTTAGGGAGTGG |
|  |  | R | ACGTTGGATGGTAGGCACCTCACAGAAATG |
|  | rs3093145 | F | ACGTTGGATGCACATGGCATTGTTTCTGGC |
|  |  | R | ACGTTGGATGAGGACTCAACGAAGGACTAC |
|  | rs3093193 | F | ACGTTGGATGGTGATGAGACTAGTGATCCC |
|  |  | R | ACGTTGGATGGCCACATACACATTGATGGG |
|  | rs3093144 | F | ACGTTGGATGAGGAGTCTCTCGTCCTTCTG |
|  |  | R | ACGTTGGATGGGGAAGAATTGTGGCAAAGG |
|  | rs3093198 | F | ACGTTGGATGCAACCCAACCGTACTCTATG |
|  |  | R | ACGTTGGATGGACATTGTAGATGGTCCAAG |
|  | rs2016503 | F | ACGTTGGATGCCACCTTTCCCCTAGAGTTC |
|  |  | R | ACGTTGGATGATCAGAGACACAGGGATTGG |
|  | rs2108622 | F | ACGTTGGATGCATCAGTGTTTTCGGAACCC |
|  |  | R | ACGTTGGATGCTCTAGGAGCCTTGGAATGG |

F= Forward, R=Reverse
