# Supplementary material for: Genes Involved in the Metabolism of Poly-Unsaturated Fatty-Acids (PUFA) and Risk for Crohn's Disease in Children & Young Adults
Source: PLoS One. 2010 Dec 20;5(12):e15672. doi: 10.1371/journal.pone.0015672 (PMC3004960; doi:10.1371/journal.pone.0015672)
Supplement: Table S2 — Associations between the CYP4F2 gene and risk for CD in Canadian children. (DOC) [file pone.0015672.s002.doc]

| **SNP** | **MODEL** | **Cases** | **Controls** | **P-value** |
| --- | --- | --- | --- | --- |
| rs1272 (C/G) | TREND | 179/661 | 203/765 | 0.86 |
|  | DOM | 165/255 | 176/308 | 0.37 |
|  | REC | 14/406 | 27/457 | 0.10 |
| rs2074900 (A/G) | TREND | 265/575 | 306/660 | 0.95 |
|  | DOM | 220/200 | 259/224 | 0.71 |
|  | REC | 45/375 | 47/436 | 0.63 |
| rs3093158 (G/A) | TREND | 252/582 | 324/638 | 0.11 |
|  | DOM | 223/194 | 267/214 | 0.54 |
|  | REC | 29/388 | 57/424 | 0.013* |
| rs2074902 (C/T) | TREND | 180/662 | 171/795 | 0.047* |
|  | DOM | 161/260 | 158/325 | 0.082 |
|  | REC | 19/402 | 13/470 | 0.14 |
| rs3093145 (A/C) | TREND | 408/434 | 466/500 | 0.93 |
|  | DOM | 308/113 | 359/124 | 0.69 |
|  | REC | 100/321 | 107/376 | 0.57 |
| rs3093193 (C/G) | TREND | 371/469 | 413/549 | 0.60 |
|  | DOM | 282/138 | 327/154 | 0.79 |
|  | REC | 89/331 | 86/395 | 0.21 |
| rs3093144 (A/G) | TREND | 140/700 | 149/801 | 0.57 |
|  | DOM | 128/292 | 138/337 | 0.64 |
|  | REC | 12/408 | 11/464 | 0.61 |

| rs3093198 (T/C) | TREND | 218/620 | 247/719 | 0.83 |
| --- | --- | --- | --- | --- |
|  | DOM | 189/230 | 209/274 | 0.58 |
|  | REC | 29/390 | 38/445 | 0.59 |
| rs2016503 (C/T) | TREND | 120/722 | 125/843 | 0.40 |
|  | DOM | 112/309 | 118/366 | 0.44 |
|  | REC | 8/413 | 7/477 | 0.59 |
| rs2108622 (T/C) | TREND | 280/550 | 290/668 | 0.10 |
|  | DOM | 236/179 | 257/222 | 0.33 |
|  | REC | 44/371 | 33/446 | 0.048* |

* significant p-value (<0.05); DOM: dominant, REC: recessive, TREND: Cochran-Armitage trend test. For this test, the numbers in the table represent the number of chromosomes that had the minor or the major allele.
